# Bactericidal Kinetics of Marine-Derived Napyradiomycins against Contemporary Methicillin-Resistant *Staphylococcus aureus*

**DOI:** 10.3390/md9040680

**Published:** 2011-04-21

**Authors:** Nina M. Haste, Lauge Farnaes, Varahenage R. Perera, William Fenical, Victor Nizet, Mary E. Hensler

**Affiliations:** 1 Skaggs School of Pharmacy and Pharmaceutical Sciences, University of California, San Diego, La Jolla, CA 92093, USA; E-Mails: nhaste@ucsd.edu (N.M.H.); wfenical@ucsd.edu (W.F.); vnizet@ucsd.edu (V.N.); 2 Center for Marine Biotechnology and Biomedicine, Scripps Institution of Oceanography, University of California, San Diego, La Jolla, CA 92093, USA; E-Mail: lfarnaes@ucsd.edu; 3 Department of Pediatrics, University of California, San Diego, La Jolla, CA 92093, USA; E-Mail: vperera@gmail.com

**Keywords:** napyradiomycin, antibiotic, antibacterial, time-kill, methicillin-resistant *Staphylococcus aureus* (MRSA)

## Abstract

There is an urgent need for new antibiotics to treat hospital- and community-associated methicillin-resistant *Staphylococcus aureus* (MRSA) infections. Previous work has indicated that both terrestrial and marine-derived members of the napyradiomycin class possess potential anti-staphylococcal activities. These compounds are unique meroterpenoids with unusual levels of halogenation. In this paper we report the evaluation of two previously described napyradiomycin derivatives, A80915A (**1**) and A80915B (**2**) produced by the marine-derived actinomycete, *Streptomyces* sp. strain CNQ-525, for their specific activities against contemporary and clinically relevant MRSA. Reported are studies of the *in vitro* kinetics of these chemical scaffolds in time-kill MRSA assays. Both napyradiomycin derivatives demonstrate potent and rapid bactericidal activity against contemporary MRSA strains. These data may help guide future development and design of analogs of the napyradiomycins that could potentially serve as useful anti-MRSA therapeutics.

## 1. Introduction

The rapid rise of methicillin-resistant *Staphylococcus aureus* (MRSA) infections worldwide has defined an urgent need for new antibiotics. MRSA is now the most common cause of skin and soft tissue infections in the United States [1], and both community-associated (CA) and hospital-associated (HA) strains of MRSA pose a great challenge to human health. This problem is compounded by the de-emphasis of antibiotic screening programs in pharmaceutical companies over the last 20 years, with only a few novel antibiotic scaffolds introduced as therapeutics over the last decade [2,3].

Most of the antibiotics used in the clinic today are derived from secondary metabolites from actinomycete bacteria of terrestrial origin [4]. This group of filamentous spore-forming bacteria have yielded a great number of today’s clinical therapies, including the commonly used antibiotics gentamicin, rifampin and vancomycin [4,5].

In 1986, a novel antibiotic class was discovered from the soil-derived organism *Chainia rubra* MG802-AF1 in Japan [6,7]. This class of meroterpenoid dihydroquinone compounds, termed napyradiomycins, was initially characterized according to their physiochemical, spectroscopic, NMR and X-ray diffraction properties [8,9]. In 1990, another actinomycete, *Streptomyces aculeolatus*, was found to produce structurally related napyradiomycins [10–14]. Early assays revealed the napyradiomycin class to have significant activities against Gram-positive bacteria including *S. aureus*, *S. epidermidis*, *Enterococcus faecalis* and *E. faecium*, *Streptococcus pyogenes* and *S. pneumoniae*, *Haemophilus influenzae*, *Bacillus anthracis*, and *Micrococcus luteus* [6,9,10,12]. Napyradiomycins, including A80915A (**1**) and A80915B (**2**), showed activity against *Clostridium difficile* and *C. perfringens* and other anaerobic bacteria; however, no activity was found against Gram-negative bacteria [10].

Over the last decade, research has focused on the discovery and characterization of natural products from marine-derived actinomycetes [5,15]. Fenical and colleagues recently isolated the marine-derived actinomycete strain, CNQ-525, that was found to be a member of the MAR 4 group of *Streptomyces* related bacteria [15–18]. This strain was also found to produce chlorine-containing meroterpenoids of the napyradiomycin class [17,18]. Through cultivation and fractionation, numerous napyradiomycin analogs were collected and initial bioactivities of these compounds were tested. Similar to the previously discovered napyradiomycins, the majority of these marine-derived derivatives were found to have both antibacterial and cytotoxic activities [17,18].

As clinical strains of MRSA become increasingly resistant to antibiotics, there is a clear need for the discovery of new antibiotics that can treat complicated infections caused by these strains. In this paper, we evaluate the antibacterial activity effects of two previously described napyradiomycin compounds (**1**) and (**2**) against a contemporary and clinically relevant panel of HA- and CA-associated strains of MRSA, two vancomycin-resistant (VRSA), and a panel of glycopeptide-intermediate *S. aureus* (GISA) strains. Additionally, we assess key pharmacological characteristics of these compounds using an *in vitro* time-kill kinetic assay. Our data illustrate that select napyradiomycins, while cytotoxic, are also potent anti-MRSA antibiotics with rapid *in vitro* time kill kinetics.

## 2. Results and Discussion

**Figure 1.**
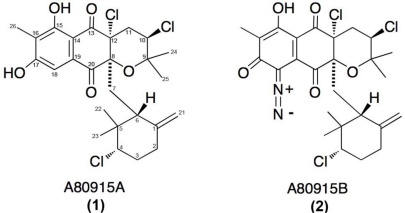
Structures of two napyradiomycins **1** and **2** isolated from the marine-derived actinomycete strain CNQ-525 and earlier from *S. aculeolatus* [10].

The actinomycte strain CNQ-525 was isolated and cultured from a marine-sediment collected at 152 m depth off the coast of La Jolla, CA, USA. This strain was subjected to 16S rRNA sequence analysis and found to be part of the MAR 4 group that is within the family Streptomycetaceae [16,17,19]. This strain has been identified as a prolific producer of halogenated MAR 4 derivatives. Two of these compounds, **1** and **2** (Figure 1), were purified from extracts of the saline cultures of strain CNQ-525.

More recently, we observed that napyradiomycins from the marine strain CNQ-525 could show activities against a test strain of MRSA and vancomycin-resistant *Enterococcus* (VRE) [17]. We investigated in detail the antibiotic properties of napyradiomycins against a panel of clinically relevant MRSA strains. Minimum inhibitory concentration (MIC) assays were preformed using broth microdilution assays and our results show that both napyradiomycins **1** and **2** possess potent anti-MRSA activities against a series of HA- and CA-MRSA strains, two VRSA strains and a series of glycopeptide-intermediate (GISA) strains (Table 1). For **1**, the MIC was ∼2 μg/mL against the CA-MRSA strains UAMS-1182 and TCH1516, both isolates of the USA300 clone currently epidemic in the U.S. Similarly, against the same strains the diazoketone **2** showed slightly more potent activities with MIC values averaging 1.5 μg/mL. Against HA-MRSA strains, both compounds exhibited MICs in the range of 1–3 μg/mL. The napyradiomycins retained their activities against the Michigan and Pennsylvania VRSA isolates as well as against GISA strains with overall MICs ranging from 0.5 to 4 μg/mL.

**Figure 2.**
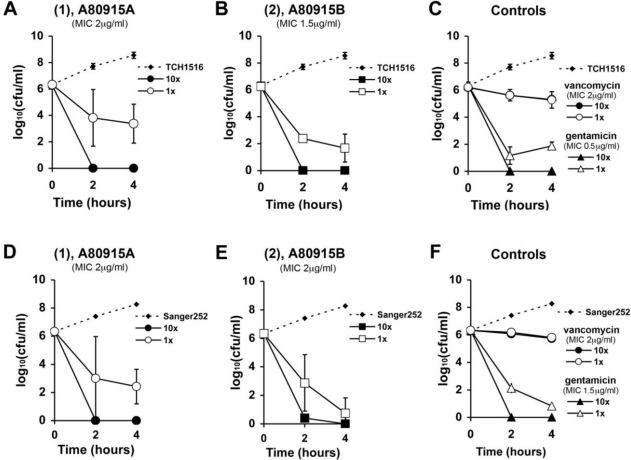
*In vitro* time-kill studies of A80915A (**1A**,**D**); A80915B (**2B**,**E**); conrols vancomycin and gentamicin (**C**,**F**) against CA-MRSA TCH1516 (**A**–**C**) and HA-MRSA Sanger 252 (**D**–**F**).

We studied the time-kill kinetics of **1** and **2** at two different concentrations, compared to vancomycin and gentamicin. *In vitro* time-kill analyses were conducted at 1× and 10× of derivative **1**, **2**, vancomycin or gentamicin control against CA-MRSA USA300 (TCH1516) (Figure 2A–C) and against the HA-MRSA Sanger 252 (Figure 2D–F). Over the first 4 h, the bactericidal activity for the two napyradiomycins **1** and **2** was more rapid than that observed for the vancomycin control and more closely paralleled the kinetics seen in the gentamicin controls (Figure 2C,F). Cultures incubated with 10× MIC of **1**, **2** or gentamicin showed complete killing at 2 h. Cultures treated with 1× MIC of **1** showed a nearly two-log reduction in bacterial counts at 4 h. The diazoketone derivative **2** showed slightly more potent and rapid killing with nearly a four-log decrease in bacterial counts at just 2 h in 1× MIC treated cultures. Vancomycin treated cultures showed time-dependent kinetics, with no difference in bacterial counts between the 10× and 1× treated cultures over the first 4 h.

Napyradiomycins **1** and **2** (A80915A and A80915B) share similar, but not identical, structural features (Figure 1). The main difference between these two derivatives is the existence of a diazoketone functionality attached to the napthoquinone core (Figure 1B). Common to both of these scaffolds is the existence of a methyl group at the C16 position and the exocyclic methylene group at C1. Interestingly, the original napyradiomycins isolated by Shiomi *et al.* [6–9] lacked this C16 methyl group, however, the compounds extracted by Fukuda *et al.*, contained this methyl feature [10].

This structural-activity relationship (SAR) data can be of importance in designing new derivatives as antibiotic candidates. For example, the C16 methyl group of A80915A (**1)** has been found to slightly reduce the cytotoxic activity in comparison to a napyradiomycin lacking the methyl group (Farnaes *et al.* [21]). Using an antiproliferative bioassay, we quantified the cytotoxicity of napyradiomycins **1** and **2** using HCT-116 human colon adenocarcinoma cells. Our results revealed a low therapeutic index for both compounds. IC_50_ values for compounds **1** and **2** were 3 μM and 500 nM, respectively. These results correlate with the studies of Fukuda *et al.*, which previously showed that in napyradiomycin derivative A80915B (**2**) the diazoketone group increased mammalian cell cytotoxicity [10]. Furthermore, our time kill analyses show that both the diazoketone derivative and **1** have effective bactericidal killing, even at 1× MIC (Figure 2). Though the diazoketone **2** may appear to have more rapid kinetics, compound **1** has similar kinetics. With this structure information in hand, it is conceivable that the napyradiomycin scaffold could be modified to contain structural moieties that reduce mammalian cell cytotoxicity yet retain or enhance anti-MRSA activities we report here.

In addition to achieving reduced cytotoxicity and enhanced antibiotic activities, further development of napyradiomycins as viable anti-MRSA drug candidates would also involve optimizing compound bioavailability. A recent study on terpenoids produced by actinomycetes compared various napyradiomycin scaffolds and revealed that the antibacterial activities were greatly diminished in the presence of horse serum [22]. We confirmed these results against the CA-MRSA USA300 strain TCH1516 using a resazurin-based color change broth microdilution MIC assay to assess for bacterial growth in the presence of serum. Our results show that in the presence of 20% normal human serum, the activity of napyradiomcyins is abolished. These results highlight a critical obstacle in the development of napyradiomycin analogs as clinical anti-MRSA therapeutics. Focused SAR studies of the napyradiomycin scaffold could conceivably identify a compound with activity in the presence of serum. Alternatively, the current napyradiomycin derivatives could be contemplated for topical applications.

Napyradiomycins have been described as novel terpenoids with promising broader therapeutic application [23]. This novel scaffold has generated recent interest in both its biosynthetic pathway [24] as well as in possible chemical syntheses [25]. The study of the molecular biosynthetic gene cluster associated with strain CNQ-525 has revealed that napyradiomycin biosynthesis involves polyketide pathways, halogenation, terpenoid biosynthetic pathways and chloronium ion-induced cyclization via a vanadium-dependent chloroperoxidase [24]. Terpenoid biosynthesis stems from a mevalonate pathway. Besides napyradiomycins, various marine-derived secondary metabolites are biosynthesized through a mevalonate pathway. For example, oxaloterpin, naphterin, terpentecin, and other terpene containing scaffolds are produced by actinomycetes with a mevalonate pathway [22,23].

## 3. Experimental Section

### 3.1. Isolation and Cultivation of CNQ-525 and Purification of Napyradiomycin Derivative **1** (A80915A) and Derivative **2** (A80915B)

The actinomycete strain CNQ-525, identified as a member of the MAR 4 clade (related to the genus *Streptomyces*) was isolated from a marine sediment collected at a depth of 152 m near La Jolla, CA, USA. Procedures detailing the isolation and cultivation of this strain and the purification of napyradiomycin compounds were previously published [17]. Briefly, strain CNQ-525 was cultivated by shaking at 30 °C for 7 days in liquid nutrient media (10 g starch, 4 g yeast extract, 2 g peptone, 1 g CaCO_3_, 5 mL of 0.8% (w/v) Fe_2_SO_4_·4H_2_O stock solution in 1 L of seawater). Cultivation was followed by extraction using an Amberlite XAD-7 resin for 6 h, filtration and extraction by acetone. The crude whole culture extract was separated by silica gel and Flash chromatography using ethyl acetate and iso-octane into ten fractions with differing polarities. Subsequently, the fractions were analyzed by LCMS, and compounds **1** and **2** were isolated by HPLC and analyzed for weight and purity prior to antibacterial assays. Compounds **1** and **2** were dissolved in DMSO, protected from light, and stored at −20 °C. Fresh samples were thawed immediately prior to each assay.

### 3.2. Bacterial Strains

A panel of HA- and CA-MRSA strains were used to evaluate the activity of napyradiomycins against contemporary and clinically relevant strains of MRSA. These strains included ATCC33591, NRS70 (N315), Sanger 252, NRS100 (COL), an MRSA clinical isolate designated #44 and a second clinical isolate designated #88, as well as UAMS1182 and TCH1516 (both USA 300 strains). In addition, the methicillin-sensitive (MSSA) strain ATCC 29213, a susceptible reference strain of *S. aureus*, was included in the panel. To further evaluate napyradiomycins against multiresistant *S. aureus* strains, we tested a panel of glycopeptide-intermediate (GISA) and two vancomycin-resistant (VRSA) strains, including HIP5836 (GISA, New Jersey, U.S.), A5940 (Hetero-GISA), PC-3 (GISA, New York, U.S.), and VRSA (Michigan, U.S.) and VRSA (Pennsylvania, U.S.). Isolates with the NRS designation were acquired via the Network of Antimicrobial Resistance in *Staphylococcus aureus* (NARSA) program (supported under NIAID/NIH contract HHSN272200700055C). The USA300 isolate was obtained from Greg Somerville at the University of Nebraska, Lincoln and originally received from Mark Smeltzer at the University of Arkansas Medical Center. The second USA300 isolate, TCH1516 (ATCC BAA-1717) and the HA-MRSA strain ATCC 33591 were obtained from the American Type Culture Collection (Manassas, VA, U.S.). Michigan and Pennsylvania VRSA strains, the panel of GISA strains, and the two MRSA clinical bacteremia isolates were obtained from George Sakoulas, MD, USA (University of California San Diego).

### 3.3. Susceptibility Testing

Minimum inhibitory concentration (MIC) assays were performed by broth microdilution according to CLSI guidelines [26]. Vancomycin (Hospira, Lake Forest, IL, USA) or gentamicin sulfate (Hospira, Lake Forest, IL, USA) were the MRSA control antibiotics. The MIC was determined to be the lowest concentration of antibiotic that inhibited bacterial growth detected at A_600_.

### 3.4. Time-Kill Analyses

Time-kill analyses were carried out as described [27]. Two strains, the CA-MRSA USA300 strain TCH1516, and the HA-MRSA Sanger 252 were prepared as described above for MIC assays to an A_600_ = 0.4 in phosphate-buffered saline in 5 mL polystyrene Falcon tubes. The tubes contained the vehicle control (DMSO), compound **1** or compound **2**, vancomycin or gentamicin at 1× or 10× MIC. The MICs against TCH1516 were 2 μg/mL, 1.5 μg/mL, 2 μg/mL and 0.5 μg/mL, respectively for compound **1**, **2**, vancomycin and gentamicin. For Sanger 252, MICs were 2 μg/mL for compounds **1**, **2**, and vancomycin and 1.5 μg/mL for gentamicin. Bacteria were added to the tubes at 5 × 10^5^ cfu/mL in 4 mL and were incubated in a shaking incubator at 37 °C. Aliquots from each tube were removed at 0, 2 and 4 h and were serially diluted in phosphate-buffered saline and plated on Todd-Hewitt agar (Hardy Diagnostics, Santa Maria, CA, USA) in the absence of antibiotics. Viable colonies were enumerated after 24 h at 37 °C with a limit of detection for the time-kill assay of 40 cfu/mL. The 0, 2, and 4 h time points were plotted to best show the early kinetics of antibiotic activities.

### 3.5. Effect of Human Serum on Antibiotic Activity

The MIC in the presence of 20% pooled human serum was completed similar to the broth microdilution assays described above except that resazurin (Sigma-Aldrich, St. Louis, MO, USA) was added at a final testing concentration of 0.675 mg/mL to assess bacterial growth. Plates were covered with foil and incubated for 24 h with shaking at 37 °C. After incubation, plates were assessed visually for color change from blue to pink to indicate bacterial growth. The MIC in the presence of 20% serum was determined to be the lowest antibiotic concentration that did not induce a blue to pink color change.

### 3.6. Antiproliferative Bioassay: Test for Cytotoxicity

Aliquots of HCT-116 human colon adenocarcinoma cells were transferred to 96-well plates and incubated overnight at 37 °C in 5% CO_2_/air. Test compounds were added to the plates in DMSO and serially diluted. The plates were then further incubated for another 72 h, and at the end of this period, a CellTiter 96 Aqueous non-radioactive cell proliferation assay (Promega) was used to assess cell viability. Inhibition concentration (IC_50_) values were deduced from the bioreduction of MTS/PMS by living cells into a formazan product. MTS/PMS was first applied to the sample wells, followed by incubation for 3 h. Etoposide (Sigma; IC_50_ = 1.549 mM) and DMSO (solvent) were used as the positive and negative controls in this assay. The quantity of the formazan product (in proportion to the number of living cells) in each well was determined by the Molecular Devices Emax microplate reader set to 490 nm wavelength. IC_50_ values were calculated using the analysis program Prism.

## 4. Conclusions

Complex meroterpenoids produced by terrestrial and marine-derived actinomycetes are promising candidates for drug discovery as antibacterial and anti-tumor agents. Early reports showed that napyradiomycins from terrestrial species had activities against many Gram-positive as well as some anaerobic bacteria [10]. Here we show that two different derivatives from this class, **1** (A80915A) and **2** (A80915B), a diazoketone derivative, have excellent antibacterial activities against a panel of contemporary HA- and CA-MRSA strains as well as clinically relevant glycopeptide-intermediate and vancomycin-resistant strains. Our results also defined rapid bactericidal kinetics of these two derivatives against MRSA. These studies highlight the napyradiomycin pharmacophore as a potential therapeutic for drug-resistant clinical strains of MRSA. Clearly, the napyradiomycins will require significant future SAR studies to reduce cytotoxic activities and enhance anti-MRSA activities and bioavailability. It would also be important to define the molecular target of these compounds.

## Figures and Tables

**Table 1.** Minimum Inhibitory Concentrations (μg/mL) of napyradiomycins against contemporary antibiotic-resistant *Staphylococcus aureus* strains.

**Strain**	**Compound 1**	**Compound 2**
*S. aureus* ATCC 29213	2	4
MRSA-ATCC33591 ^a^	1–2	2
NRS70 (N315) ^a^	1	2
Sanger 252 ^a^	1–2	1
NRS100 (COL) ^a^	1	2
MRSA clinical bacteremia isolate c-44 ^a^	3	1.5
MRSA clinical bacteremia isolate c-88 ^a^	1.5–3	1.5
MRSA USA300 (UAMS1182) ^b^	1.5–3	1–3
MRSA USA300 (TCH1516) ^b^	2	1–2
VRSA (Michigan Isolate)	1–2	2–4
VRSA (Pennsylvania Isolate)	1–2	4
GISA (HIP5836) (New Jersey) ^c^	0.5	1
GISA (PC-3) (New York) ^c^	0.5	1
Hetero-GISA A5940 ^c^	1	4

a HA-MRSA strains;

b CA-MRSA strains;

c Sakoulas *et al.* [20].
